# Knowledge and practice of personal protective measures during the COVID-19 pandemic: A cross-sectional study in Saudi Arabia

**DOI:** 10.1371/journal.pone.0243695

**Published:** 2020-12-11

**Authors:** Abdulrahman S. Bazaid, Abdu Aldarhami, Naif K. Binsaleh, Subuhi Sherwani, Omar W. Althomali

**Affiliations:** 1 Department of Clinical Laboratory Sciences, College of Applied Medical Sciences, University of Ha’il, Ha’il, Saudi Arabia; 2 Department of Clinical Laboratory Sciences, Turabah University College, Taif University, Turabah, Saudi Arabia; 3 Department of Biology, College of Sciences, University of Ha’il, Ha'il, Saudi Arabia; 4 Department of Physiotherapy, College of Applied Medical Sciences, University of Ha’il, Ha’il, Saudi Arabia; Health Services Academy Islamabad Pakistan, PAKISTAN

## Abstract

The severe acute respiratory syndrome coronavirus-2 (SARS-CoV-2) is an emergent infectious pathogen causing an acute respiratory disease called corona virus disease 2019 (COVID-19). Virus transmission may occur by contact, droplet, airborne or *via* contaminated surfaces. In efforts to effectively control the COVID-19 outbreak, the world health organization (WHO) and the Saudi Ministry of Health (MOH) have advised the public to practice protective measures to reduce transmission of the virus and reduce incidence of infection. These measures include hand washing, wearing masks and gloves and avoidance of touching the face with unwashed hands. The current study aimed to investigate knowledge and adherence of the Saudi population to these protective actions during the pandemic. After determining the required sample size using power analysis, a cross-sectional online self-reported survey of 5105 Saudi residents was conducted between 25^th^ March to 17^th^ April 2020 to evaluate public knowledge of COVID-19. Participants were all aged 18 years or above, Arabic speakers and residents of Saudi Arabia. Scores were calculated based on knowledge and adherence of the individuals to protective measures. About 90% of participants exhibited a high level of knowledge (scored 2/2) and practice (scored > 3/6) in relation to hand hygiene and wearing gloves and masks. Practice scores were positively associated with females and individuals with high income. Lower practice scores were linked to youth and residents of the northern and western regions of the Kingdom. Over two thirds of participants preferred hand washing to alcohol disinfection, and the frequency and performance of hand washing improved during the pandemic for more than half of respondents. Overall, the findings reflected high public knowledge of SARS-CoV2 transmission routes and adherence to personal protective measures. However, public awareness campaigns with an emphasis on the youth and individuals with low education and income are required to improve overall practice.

## Introduction

A novel strain of coronavirus was first reported in Wuhan, China, and has since spread internationally causing a pandemic [1]. This viral strain was designated as severe acute respiratory syndrome coronavirus 2 (SARS-CoV2), the causative agent of coronavirus disease 2019 (COVID-19), which carries a high infectious rate in humans [1]. The infectious nature of the disease, rising numbers of cases, daily mortalities and a lack of therapeutics have led to a healthcare crisis of epic proportions. At the time of writing this report, the pandemic has led to more than 13 million cases and 593000 deaths worldwide [2]. In Saudi Arabia 253,349 cases have been registered with 2523 mortalities recorded till 20^th^ July 2020 [3].

Early studies suggested that SARS-CoV2 is transmitted directly through respiratory droplets from infected individuals or contaminated materials [4]. However, as a greater understanding of the virus is achieved, the routes of transmission have been widened by the WHO to include possible human-human contact, droplet, airborne, fomite, fecal-oral, bloodborne, mother-to-child and animal-to-human transmission [5]. The virus has an incubation period of up to 14 days in infected individuals either with common symptoms, including fever, cough and shortness of breath, or without signs of the infection (asymptomatic) [6]. The ability of SARS-CoV2 to cause severe complications in a relatively short span of time in a cross-section of infected individuals, with devastating repercussions ranging from acute pneumonia, respiratory distress syndrome, heart failure, cytokine storm and multi-organ dysfunction present a unique challenge and burden to health care facilities around the world [7]. Hence, transmission of the SARS-CoV2 from asymptomatic carriers is one of the major preventative challenges for controlling this viral infection [8].

In response to the declaration of global pandemic on 11 March 2020 [9], extreme prevention methods were adopted by countries around the world. The public were instructed by the WHO to seek information about COVID-19 solely from well-trusted sources (e.g. national public health authorities) and to practice protective measures, including social distancing, hand hygiene and refraining from touching the eyes, nose and mouth with unwashed hands [10, 11]. It was previously reported that raising public awareness about the Middle East Respiratory Syndrome (MERS) and the importance of hand washing had shown positive outcomes in containing previous MERS outbreaks in Saudi Arabia [12]. Hence, building on previous experience, efforts to mitigate the novel SARS-CoV2 outbreak were made by the Ministry of Health (MOH), Saudi Arabia by establishing various campaigns. These aimed to increase public awareness and knowledge about SARS-CoV2 transmission patterns, COVID-19 infection symptoms such as fever, dry cough, fatigue and related precautionary measures [13].

Recent studies conducted within the Kingdom, reported high levels of awareness among the Saudi population in relation to social distancing as a preventive step to control the spread of SARS-CoV2, and adherence to this approach was determined to be acceptable in containing the spread of the virus [14, 15]. Information about the overall knowledge and practice of other protective measures by the Saudi population would help to address the gaps between perception and practice and would allow decision makers and public health authorities to gauge national awareness and adherence to these preventative measures in order to address any deficiencies by suitable targeted means and campaigns. Thus, the aim of this study was to evaluate the knowledge, awareness and compliance of the Saudi public in relation to SARS-CoV2 preventative measures such as hand washing, wearing masks and gloves and avoidance of hand shaking and touching the face. Additionally, behavioral changes of Saudi individuals during the COVID-19 pandemic regarding the frequency and method of hand washing was assessed.

## Methods

### Study design and samples

An epidemiological investigation using a cross-sectional study design was conducted between March 25^th^ and April 17^th^, 2020 using an online self-reported questionnaire to evaluate public knowledge of COVID-19 as well as the adherence of the Saudi population to precautionary measures when leaving the home. Participants recruited previously [14] to evaluate their knowledge and commitment to social distancing (staying-at-home), were instructed to answer further questions related to wearing masks and gloves and washing hands. The questionnaire was written in Arabic and distributed online through different social media platforms (Twitter, WhatsApp and Snapchat) to ensure wide dissemination to the target population (Saudi residents aged 18 years and above) and a good coverage of respondents from different sociodemographic backgrounds. Participants could easily access the questionnaire and responses once collected were exported and analysed. The sample size was calculated using the G-Power software, version 3.0.10, based on multiple regression test with an alpha error of 5%, a power of 95% and allowing 32 predictors to be included in the model, to 5075 participants.

### Measurement and scoring system

The self-reported questionnaire was created according to the Saudi MOH and WHO guidelines to measure the knowledge and commitment of the Saudi public to protective measures, including hand hygiene, wearing masks and gloves and avoiding hand shaking [10, 16]. To ensure the accuracy of the survey's questions and their relatedness to the objectives of the study, questions were authenticated and validated by five volunteered multidisciplinary experts. In addition, language and clarity of the survey was assessed by piloting with 20 people from the public belonging to different age groups and genders. Obtained feedback from both validation groups has led to the final version (version for the study) with clear, concise and easy to understand questions that address all objectives of this study.

The introductory part of the questionnaire encompassed the purpose and objectives of the study and consent information to ensure voluntary participation in the study as well as participant anonymity and confidentiality. The survey was broadly divided into three sections (S1 File). The first section consisted of questions associated with sociodemographic information of the participants (age, gender, marital status, education, work status, monthly income, and geographical region of residence). The second section of the questionnaire (Yes/No questions) was designed to investigate the knowledge of the participants about the likelihood of SARS-CoV2 transmission through contact with contaminated surfaces and awareness about the correct method (five steps) of hand washing [11]. Furthermore, based on their knowledge, respondents were asked to select the most effective approach from a list of preventive actions as a controlling step against the spread of the COVID-19 pandemic. The listed actions were as follows: washing hands before touching the face (eyes, nose, or mouth), taking vitamins to boost immunity, using a mouthwash, receiving the influenza vaccine or avoiding contact with patients of chronic diseases. The final section of the survey was made up of six questions to evaluate the practice of protective measures among participants when leaving the home. Questions of this section were included to evaluate adherence of the Saudi population to recommended precautions, including the duration of hand washing (40 seconds), avoiding hand shaking, and wearing gloves and facial masks. The estimated time taken to complete questions related to this study was approximately 5 minutes.

To evaluate the knowledge and practice of participants when leaving the home, a gradient scoring scale between 0 and 2, and 0 to 6, was followed respectively (Table 1). Each response provided by the participants to questions of the survey was given a designated score; one point was allocated to the most appropriate answer, while the least appropriate responses and those with an indication of absent practice or an incorrect answer were scored zero points. Additionally, a gradient scoring system was followed for questions measuring the frequency of application, in which responses with the word always, often or sometimes were scored as one (1), half (0.5) or a quarter (0.25) of a point, respectively. Six points was the full score that an individual with perfect adherence to protective actions when leaving the home could obtain in this study. Participant scored >1 or >3 considered to have high knowledge or practice, respectively.

**Table 1:** Scoring scale and interpretation guidelines for responses to questions that are assessing knowledge and practice of participants.

Questions	Minimum and maximum score	Score guide
**Knowledge**		
• Do you think that SARS-CoV2 can be transmitted through contact with contaminated surfaces?	0–2	> 1 consider high knowledge
• Do you know there is a correct way (five steps) to washing your hands?		
**Practice**		
• Do you avoid shaking hands?	0–6	> 3 consider high practice
• Do you wash hands or use alcohol gel when returning home?		
• How often do you follow the WHO recommendations on hand washing method?		
• When you leave home, do you wear gloves?		
• When you go to the supermarket, do you use facial masks provided by the supermarkets?		
• Since the beginning of the COVID-19 pandemic, I changed:		

### Statistical analysis

Statistical analyses were performed using Statistical Package for the Social Sciences (SPSS) software version 25.0 (IBM Corp, Armonk, NY). Descriptive statistical analysis was applied to calculate numbers of participants, percentages, the mean, standard deviation (SD) and the median in relation to variables describing knowledge and practice. Knowledge questions were analysed using Chi-square test (more than 2 groups) or Fisher’s Exact test (2 groups) to demonstrate any significant differences between responses in relation to demographic characteristics [17]. The two knowledge questions were added together and treated as ordinal data on scale out of 3 (0–2). To test the significant difference in the total knowledge scores between the groups Mann-Whiteney U test (2 groups) or Kruskal-Wallis H test (more than 2 groups) were used. Binomial logistic regression was used to identify factors related to low knowledge in each question. Log odds value was used to show increase of probability of having low knowledge with each one unit increase in the indented outcome.

To investigate the difference between each demographic characteristic in total practice score, independent t-test (2 groups) and one-way analysis of variance (ANOVA), for normally distributed data, were conducted. Otherwise Mann-Whitney U test (2 groups) or Kruskal-Wallis H test (more than 2 groups) were used. [17, 18]. Multiple linear regression (Stepwise method) was applied to identify demographic characteristics associated with total practice scores. Odds ratios (ORs), unstandardized regression coefficients (cut-off probability for adding and removing the variable were 0.05 and 0.1, respectively) and 95% confidence intervals (CIs) were calculated to indicate find out any significant (p < 0.05) relationships between variables.

### Ethical approval

Ethical approval for the study protocol, questions and consent statement was granted by the Ethics Committee at the University of Ha’il with the reference number H-2020-80. Individuals willing to participate in this survey needed to click the ‘Continue’ button and only then would be directed to the questionnaire.

## Results

### Sociodemographic characteristics

A total of 5105 questionnaires were completed online. The majority of respondents were female (58.4%). The respondents were distributed according to age as 18–27 years, 28–37 years, 38–47 years and > 47 years. The majority of respondents were young (66.3% aged below 37 years) with the maximum in the age group of 28–37 years old. Over half of the respondents (60.9%) were married, had attained tertiary education (55.8% with a bachelor degree) or above and were urban residents (93.8%) of Saudi Arabia. A majority of respondents self-reported a minimum monthly income of ≤ 3000 SR. Conversely, a minimum number of respondents (7%) reported the maximum monthly income (≥ 20000 SR). About 45% of respondents reported themselves as being employed in government or private sectors. A little over half (51%) of participants were from West of Saudi Arabia. Social demographic characteristics of participants are detailed in Table 2.

**Table 2:** Sociodemographic characteristics of the participants.

Demographic characteristics		Number of participants
		(% of participants)
**Gender**	Male	2125 (41.6)
	Female	2980 (58.4)
**Age group**	18–27 years old	1500 (29.4)
	28–37 years old	1884 (36.9)
	38–47 years old	1059 (20.7)
	Above 47 years	662 (13.0)
**Marital status**	Single	1779 (34.8)
	Married	3107 (60.9)
	Divorced	219 (4.3)
**Educational level**	Secondary and below	1219 (23.9)
	Diploma	525 (10.3)
	Bachelor	2851 (55.8)
	Master and PhD	510 (10.0)
**Monthly income**	Less than 3000 SR	2086 (40.9)
	Between 3000–5999 SR	703 (13.8)
	Between 6000–10999 SR	734 (14.4)
	Between 11000–15999 SR	835 (16.4)
	Between 16000–20000 SR	392 (7.7)
	Above 20000 SR	355 (7.0)
**Employment**	Unemployed	1515 (29.7)
	Student	695 (13.6)
	Employed	2310 (45.2)
	Entrepreneur	38 (0.7)
	Retired	307 (6.0)
	Others	240 (4.7)
**Geographical regions**	West	2603 (51.0)
	Middle	1281 (25.1)
	East	268 (5.2)
	North	655 (12.8)
	South	298 (5.8)
**Do you live in**	City	4787 (93.8)
	Village	318 (6.2)

SR stands for Saudi Riyals (1SR = 0.27 United State Dollars).

### Knowledge about transmission of COVID-19 and hand washing techniques

The majority of respondents (89.4%) showed a high overall knowledge (scored 2 out of 2) about hand washing method and transmission of SARS-CoV2 through contaminated surface. However, about 10% of participants have answered one question correctly (scored 1 out of 2). When comparisons were made based on age, a significant reduction in knowledge was observed in individuals aged between 18–27 and 28–37 years compared to 38–47 age group. Moreover, those with secondary degree or below and low income (<3000 SR) showed significantly low knowledge compared to higher degree holders and those with high income (above 20000 SR), respectively (S1 Table).

Multiple logistic regression of each knowledge question showed that participants with lower levels of education (secondary and below, OR: 0.606, *p* = 0.001; diploma holders OR: 0.627, *p* = 0.031), and low income (<3000 SR, OR: 0.444, *p* < 0.001 and 3000–5999 SR, OR: 0.599, *p* = 0.02), were less likely to select the correct answer in relation to SARS-CoV2 transmission from surfaces. Also, residents of the western part of the Kingdom were more likely to choose the correct answer (OR: 1.402, *p* = 0.014). (Table 3). With respect to the second question about the correct five-step method of hand washing, females (OR: 2.835, *p* < 0.001), participants aged 38–47 years (OR: 1.39, *p* = 0.035), employed individuals (OR: 1.295, *p* = 0.043), those with the highest reported income (>20000 SR, OR: 1.806, *p* = 0.028), and residents of the middle region (OR: 1.402, *p* = 0.023) of the Kingdom were more likely to choose the correct answer to this question. However, the wrong answer was associated with participants with a secondary degree or lower (OR: 0.56, *p* < 0.001) and residents of the northern regions (OR: 0.691, *p* = 0.018) of the Kingdom (Table 3).

**Table 3:** Binomial logistic regression analysing association between demographic characteristics and provided responses questions that are evaluating knowledge in relation to hand washing and transmission of SARS-CoV2 through contaminated surfaces.

	OR	*p* value		OR confidence interval (95%)		
				Lower bound		Upper bound
**Question 1: Do you think that SARS-CoV2 can be transmitted through contact with contaminated surfaces?**						
Education (secondary and below)	0.606	0.001*		0.45		0.816
Education (diploma)	0.627	0.031*		0.41		0.959
Income (Less than 3000 SR)	0.444	<0.001*		0.324		0.607
Income (3000–5999 SR)	0.599	0.02*		0.39		0.922
Region (West)	1.402	0.014*		1.07		1.837
Constant	35.707	<0.001*				
**Question 2: Do you know there is a correct way to washing your hands?**						
Gender (female)	2.835		<0.001*		2.214	3.63
Age (38–47 years)	1.39		0.035*		1.023	1.888
Education (secondary and below)	0.56		<0.001*		0.44	0.713
Income (Above 20000 SR)	1.806		0.028*		1.067	3.058
Employment- (employed)	1.295		0.043*		1.008	1.663
Region (middle)	1.402		0.023*		1.047	1.879
Region (north)	0.691		0.018*		0.509	0.937
Constant	7.667		<0.001*			

OR, odds ratios

* statistical significance, SR stands for Saudi Riyals (1SR = 0.27 United State Dollars).

According to the responses of participants, the best preventive method to control the spread of SARS-CoV2 was determined to be washing hands before touching the face (97.1%) followed by taking vitamins to boost immunity (20.5%). Using a mouthwash, vaccination against the flu and avoiding contact with patients suffering from chronic diseases were the least likely to be selected as preventive steps by the participants (Table 4).

**Table 4:** Responses of participants to the question asking about the effective way to prevent/control the spread of SARS-CoV2.

Question	Choices	Total number of responses (%*)
**Which of the following can be defined as an effective way to prevent infection with SARS-CoV2?**	Washing hands before touching the eyes, nose, and mouth	4958 (97.1)
	Taking vitamins to boost immunity	1046 (20.5)
	Using a mouthwash	214 (4.2)
	Influenza vaccine	170 (3.3)
	Avoiding contact with those with chronic diseases	59 (1.2)

* Participants were permitted to select more than one option for this question.

### Practice of preventive measures when leaving the home

The majority of participants (90%) exhibited a high level of practice (scored > 3 out of 6) in relation to hand hygiene and wearing gloves and masks (Tables 5 and 6), with one in seven of this group scored 6 out of 6, i.e. 100% implementation. Multiple linear regression analysis (Table 7) revealed that practice scores were positively associated with females (β: 0.374, *p* < 0.001), residents in cities (β: 0.224, *p* < 0.001) and individuals with high income (>20000 SR, β: 0.174, *p* = 0.001). In contrast, low scores were linked to the age groups [18–27 years (β: -0.283, *p* < 0.001) and 28–37 years (β: -0.17, *p* < 0.001)] and regions [living in northern (β: -0.288, *p* < 0.001) or western (β: -0.071, *p* = 0.012)] of the Kingdom.

**Table 5:** Total scores of responses for questions in relation to hand hygiene and wearing gloves/masks when leaving the home during the COVID-19 pandemic.

Demographic characteristics		Mean	Standard deviation	Median	*p* value
**Gender**	Male	4.58	1.04	4.75	<0.001*
	Female	4.91	0.87	5.00	
**Age group**	18–27 years	4.64	1.01	5.00	<0.001*
	28–37 years	4.75	0.95	5.00	
	38–47 years	4.90	0.89	5.00	
	Above 47	4.93	0.92	5.00	
**Marital status**	Single	4.69	0.99	5.00	<0.001*
	Married	4.81	0.94	5.00	
	Divorced	5.01	0.83	5.00	
**Educational level**	Secondary and below	4.78	0.97	5.00	0.791
	Diploma	4.77	0.98	5.00	
	Bachelor	4.76	0.96	5.00	
	Master and PhD	4.81	0.92	5.00	
**Income**	Less than 3000 SR	4.77	0.98	5.00	0.061
	3000–5999 SR	4.83	0.90	5.00	
	6000–10999 SR	4.73	0.94	5.00	
	11000–15999 SR	4.74	0.98	5.00	
	16000–20000 SR	4.73	0.97	5.00	
	Above 20000 SR	4.89	0.93	5.00	
**Employment**	Unemployed	4.87	0.90	5.00	<0.001*
	Student	4.61	1.06	5.00	
	Employed	4.74	0.96	5.00	
	Entrepreneur	4.70	0.96	5.00	
	Retired	4.94	0.94	5.00	
	Other	4.78	0.99	5.00	
**Geographical regions**	West	4.79	0.96	5.00	<0.001*
	Middle	4.87	0.89	5.00	
	East	4.90	0.94	5.00	
	North	4.53	1.00	4.50	
	South	4.61	1.07	5.00	
**Do you live in**	City	4.79	0.95	5.00	<0.001*
	Village	4.53	1.05	4.50	
**Overall**		4.77	0.91	5.00	

* statistical significance, SR stands for Saudi Riyals (1SR = 0.27 United State Dollars).

**Table 6:** Responses to questions assessing practice of Saudi population to the recommended protective methods during the COVID-19 pandemic.

Questions	Choices	Total number of responses (%)
Do you avoid shaking hands?	Yes, always	3251 (63.7)
	Yes, often	926 (18)
	Yes, sometimes	703 (13.8)
	No, I do not think it can reduce the spread of the virus	91 (1.8)
	No, because it means disrespect to others	65 (1.3)
	No, because the society does not like it	69 (1.4)
Do you wash hands or use alcohol gel when returning home?	I wash my hands with soap and water for 40 seconds	3469 (68)
	I wash my hands with soap and water for less than 40 seconds	662 (13)
	I wash my hands with soap and use alcohol gel	369 (7.2)
	I use alcohol gel for 5 seconds	120 (2.4)
	I use alcohol gel for 20 seconds	117 (2.3)
	Other	325 (6.3)
	Neither wash my hands nor use gel	43 (0.8)
How often do you follow the WHO recommendations on hand washing method?	Always	3497 (68.5)
	Sometimes	628 (12.3)
	I started after reports of cases in Saudi Arabia	951 (18.6)
	I do not follow these recommendations	29 (0.6)
When you leave home, do you wear gloves?	Yes, always	1856 (36.4)
	Yes, sometimes	940 (18.4)
	No	932 (18.2)
	Other or I do not leave the home	1377 (27)
When you go to the supermarket, do you use facial masks provided by the supermarkets?	Yes, always	2654 (52)
	Yes, sometimes	466 (9.1)
	No, because masks are not provided	241 (4.7)
	No, I do not want to use them	181 (3.5)
	Other or I do not leave the home	1563 (30.7)
Since the beginning of the COVID-19 pandemic, I changed:	Frequency and method of hand washing	2682 (52.5)
	Frequency of hand washing	1307 (25.6)
	Hand washing method	756 (14.8)
	Nothing changed	360 (7.1)

**Table 7:** Multiple linear regression analysing the associations between demographic characteristics and calculated scores of participants in relation to hand hygiene and wearing gloves/masks when leaving the home.

	Β	SE	*p* value	OR confidence interval (95%)	
				Lower bound	Upper bound
Gender (female)	0.374	0.027	<0.001*	0.321	0.428
Age (18–27 years)	-0.283	0.034	<0.001*	-0.35	-0.217
Region (North)	-0.288	0.042	<0.001*	-0.371	-0.205
Age (28–37 years)	-0.17	0.031	<0.001*	-0.231	-0.108
Resident (City)	0.224	0.054	<0.001*	0.118	0.33
Income (<20000SR)	0.174	0.053	0.001*	0.07	0.279
Region (West)	-0.071	0.028	0.012*	-0.127	-0.016
Constant	4.551	0.06	<0.001*	4.433	4.67

β, slope of the regression; SE, standard error

* statistical significance. SR stands for Saudi Riyals (1SR = 0.27 United State Dollars).

In response to preventative measures imposed by authorities to minimize viral spread, participants were asked to self-report behavior. Generally, more than half of participants responded similarly to questions for practicing the recommended protective measures, except the question of wearing gloves when going out, where the percentage was found to be 36.4% (Table 6). Approximately 82% of participants stopped shaking hands when greeting others and most of the participants (68%) fulfilled the WHO recommendations of washing their hands when returning home. However, 18% of participants adhered to the recommend method of washing hands only when confirmed cases of COVID-19 started to be reported in the Kingdom of Saudi Arabia. Both the frequency and method of washing hands improved during COVID-19 pandemic for more than 50% of the participants. In the context of using personal protective equipment, more than half of the participants wore gloves and facial masks when leaving the home.

## Discussion

The onslaught on a new infectious disease COVID-19 has proved challenging for health care infrastructures worldwide. In the absence of proven therapeutics, a well-tested vaccine, and with the number of new infections still growing at an alarming rate globally, preventative steps are essential to break the chain of transmission of the virus and control infection rates. Evidence suggests that public awareness and preventive behaviors are critical in the control of epidemics. Hence, critical steps have been taken by health authorities to consistently disseminate accurate information to control and manage public behavior [19]. The WHO and the Saudi MOH advised the general public to apply certain precautionary measures as controlling steps for the COVID-19 pandemic, including washing the hands, avoiding hand shaking, avoid touching the face with unwashed hands and ensure wearing masks [10, 16]. These steps if diligently followed by the public, could lead to a decrease in the incidence of infection in the general population and reduce the risk of transmission. Therefore, this study aimed to determine the level of knowledge and awareness of the Saudi population in relation to transmission of SARS-CoV2 and estimate practice of recommended protection and preventative measures on a day to day basis and when leaving the home during the current COVID-19 outbreak. Various socioeconomic characteristics were considered in this study to understand conditions influencing reported outcomes to evaluate the gaps in knowledge, understanding and behavior of the general public in adapting to and following recommended mitigation measures.

The majority of respondents in our study displayed a high level of knowledge and adherence to suggested preventative measures. Several similar studies have also indicated high levels of knowledge of COVID-19 among the public [20, 21]. The majority of participants in our survey were young. This trend can be explained by the demographic data reported by the General Authority for Statistics, Kingdom of Saudi Arabia [22] as well as the tendency of a relatively young population to regularly use smartphones and social media. The level of knowledge about SARS-CoV2 transmission through contaminated surfaces varied among participants. For instance, residents of the western region of the Kingdom showed higher levels of knowledge compared to their counterparts from other regions. It is not surprising that people from low socioeconomic backgrounds and with lower levels of education presented lower knowledge about COVID-19 transmission. Similar trends have been reported in earlier studies [23–25] and may be due to difficulties of either reaching or understanding published information.

An overwhelmingly large majority of participants in this study (97%) demonstrated high knowledge of hand hygiene and washing hands before touching their face to reduce transmission. Adherence of the public to hand washing is probably one of the most cost-effective ways to control the spread of SARS-CoV2 in communities [26]. Knowledge of the participants regarding this recommended method of washing the hands and the possibility of SARS-CoV2 transmission through contaminated surfaces was determined to be high (89%). In line with previous studies, females [23–25] and individuals aged between 38 and 47 years showed greater awareness about the correct manner of washing their hands in comparison with other participants. Significantly, high levels of knowledge of COVID-19 transmission and preventive measures to tackle its spread may be the outcome of multiple awareness campaigns conducted by the MOH through various mediums [13]. This is because the majority of participants, as indicated by a previous study, were using the Saudi MOH as their main source of information about COVID-19 and related preventive steps [14].

Residents of the northern region appeared to be less aware about the importance of washing their hands, which is in agreement with an earlier study where residents of Aljouf, north of the Kingdom, exhibited only moderate understanding about the incubation period and clinical signs of MERS [27]. Adherence to preventive measures such as hand hygiene and wearing gloves and masks in public by the residents of northern parts of the Kingdom may be less than other parts since confirmed cases of COVID-19 only started to be reported 4 weeks after the first reported cases in the country [28].

Almost all participants (90%) exhibited high practice scores (> 3 out of 6) in relation to adherence to protective measures when leaving the home, although 14% of those participants scored 6 (100% implementation). Good implementation of these personal protective measures should compensate for the acceptable levels of social distancing among the Saudi population [14]. Females, individuals with high income and urban residents were significantly associated with high scores in relation to the frequency and method of washing the hands and wearing personal protective equipment in comparison with their counterparts. This finding is in line with previous studies conducted on hand hygiene and behavioral changes of various nations towards protective measures, which reported high practice by females [20, 29, 30], urban residents [31] and individuals with high socioeconomic status [20, 29]. A previous study reported that two third of Arab individuals were of the view that it was unnecessary to adhere to the practice of wearing facial masks and gloves by children and young people when leaving the home [32]. However, many children and young people have reportedly been infected by SARS-CoV2 worldwide, including Saudi Arabia [28, 33]. Generally, people from these age groups are more mobile, active and less affected by the virus. Thus inadequate or non-compliance of protective measures may lead to a high chance of spreading infection within communities, especially within multigenerational families. This might explain the negative correlation between practice of protective measures and younger participants (below 38 years old) that was revealed in the current study. Thus, public awareness campaigns and community engagement strategies focusing on the effective communication of risks as well as protection and prevention measures, especially in environments frequented by the youth such as cafes and sports centers, are recommended for spreading awareness amongst low practice groups.

The reasons behind poor compliance of various preventive measures were investigated. Participants who did not avoid hand shaking reported reluctance due to feelings of disrespect towards others or the belief that such a step was ineffective in controlling the spread of SARS-CoV2. Hand shaking is strongly linked with cultural values in many nations, including the Arab culture [34], and difficulties associated with avoidance of such customs although understandable must be corrected by increasing awareness. Local health authorities should address such awareness deficiencies to effectively control the dissemination of COVID-19 among communities.

Worldwide public were instructed to disinfect hands with soap for at least 40 seconds or with alcohol gel for 20 seconds when returning home [8]. One in seven participants in this study reported disinfecting their hands using soap and water or alcohol gel for less than the recommended time, which may lead to unsuccessful disinfection. Nearly 7% of participants reported that they did not wash their hands when returning home or that they did not follow the recommended method. In addition, almost one in five respondents reported starting to apply these recommendations only when cases of COVID-19 were confirmed in Saudi Arabia.

Generally, the COVID-19 pandemic has been linked with behavioral changes in the Saudi population in relation to adoption of preventive measures as a routine [24, 25]. More than half of the participants of this study reported improved hand hygiene, whereas the daily frequency of hand washing increased by 25% of the participants. Good practice of hand hygiene and regular use of masks and gloves in the community has many health benefits, including limiting prevalence of many infectious diseases targeting various body systems, such as the skin, gastrointestinal tract and respiratory system [35]. The observed commitment and behavioral changes of the Saudi public in relation to adoption of safety measures would suggest implications for limiting the spread of SARS-CoV2 infection.

## Conclusion

Adherence of public to the advised personal protective measures by the WHO, including hand hygiene and wearing gloves and masks, is crucial to control the COVID-19 pandemic. This study showed that the Saudi population has a relatively high knowledge and implementation of hand hygiene and wearing facial masks and gloves. Increased awareness and practice were associated with females, high socioeconomic status and high levels of education. Low practice of protective measures was significantly associated with youth (below 37 years old) and residents of the northern part of the Kingdom as well as residents with a lower level of education or income. Tailored education programs and emphasis by public health authorities on sustained compliance of protective measures by the public is necessary for individuals with low practice to improve their overall practice of personal protective measures.

### Limitations of this study

The practice of hand hygiene is a socially desirable behavior. Therefore, in a self-reported study, respondents may over-report practices related to hand washing and hence lead to inflated results. Non-obtrusive monitoring studies may yield more unbiased results and may be a better representation of actual participant behavior. However, such a mode of data collection would be ill advised in the middle of the current pandemic.

## Supporting information

S1 File Designed questionnaire in English and Arabic. (PDF)

S1 Table Mean rank of total scores in regards to knowledge questions. (PDF)
