# Supplementary material for: Knowledge and practice of personal protective measures during the COVID-19 pandemic: A cross-sectional study in Saudi Arabia
Source: PLoS One. 2020 Dec 11;15(12):e0243695. doi: 10.1371/journal.pone.0243695 (PMC7732079; doi:10.1371/journal.pone.0243695)
Supplement: S1 File — (PDF) [file pone.0243695.s001.pdf]

S1: Designed questionnaire in English and Arabic

Gender

A. Male

B. Female

Age group

A. 18-27 years

B. 28-37 years

C. 38-47 years

D. Above 47

Marital status

A. Single

B. Married

A. Divorced

Educational level

A. Secondary and below

B. Diploma

C. Bachelor

D. Master and PhD

Monthly income

A. Less than 3000 SR

B. 3000-5999 SR

C. 6000-10999 SR

D. 11000-15999 SR

E. 16000-20000 SR

F. Above 20000 SR

Employment

A. Unemployed

B. Student

C. Employed

D. Entrepreneur

E. Retired

F. Other

Which of the following geographical regions are you from:

A. West

B. Middle

C. East

D. North
E. South

Your residency in a

A. City
B. Village

Which of the following can be defined as an effective way to prevent infection with SARS-CoV2?

A. Washing hands before touching the eyes, nose, and mouth
B. Taking vitamins to boost immunity
C. Using a mouthwash
D. Influenza vaccine
E. Avoiding contact with those with chronic diseases

Do you think that SARS-CoV2 can be transmitted through contact with contaminated surfaces?

A. Yes
B. No

Do you know there is a correct way (five steps) to washing your hands?

A. Yes
B. No

Do you avoid shaking hands?

A. Yes, always
B. Yes, often
C. Yes, sometimes
D. No, I do not think it can reduce the spread of the virus
E. No, because it means disrespect to others
F. No, because the society does not like it

Do you wash hands or use alcohol gel when returning home?

A. I wash my hands with soap and water for 40 seconds
B. I wash my hands with soap and water for less than 40 seconds
C. I wash my hands with soap and use alcohol gel
D. I use alcohol gel for 5 seconds

E. I use alcohol gel for 20 seconds
F. Neither wash my hands nor use gel
G. Other

How often do you follow the WHO recommendations on hand washing method?

A. Always
B. Sometimes
C. I started after reports of cases in Saudi Arabia
D. I do not follow these recommendations

When you leave home, do you wear gloves?

A. Yes, always
B. Yes, sometimes
C. No
D. Other or I do not leave the home

When you go to the supermarket, do you use facial masks provided by the supermarkets?

E. Yes, always
F. Yes, sometimes
G. No, because masks are not provided
H. No, I do not want to use them
I. Other or I do not leave the home

Since the beginning of the COVID-19 pandemic, I changed:

A. Frequency and method of hand washing
B. Frequency of hand washing
C. Hand washing method
D. Nothing changed

الجنس
ذكر

أنثى

الفئة العمرية
١٨-٢٧ سنة
٢٨-٣٧ سنة
٣٨-٤٧ سنة
أكبر من ٤٧ سنة

الحالة الاجتماعية
أعزب
متزوج
مطلق

مستوى التعليم
المرحلة المتوسطة او اقل
المرحلة الثانوية
دبلوم
جامعي
ماجستير
دكتوراه

الدخل الشهري
اقل من ٦ آلاف ريال سعودي
من ٦ -١٠ آلاف ريال سعودي
١١ -١٥ ألف ريال سعودي
١٦-٢٠ ألف ريال سعودي
أكثر من ٢٠ ألف ريال سعودي

الوظيفة
موظف
متقاعد
رائد اعمال
طالب
اخرى

المنطقة الجغرافية التي تسكن بها
المنطقة الوسطى
المنطقة الغربية
المنطقة الشرقية
المنطقة الشمالية
المنطقة الجنوبية

مكان اقامتك او سكنك في
إحدى مدن المنطقة
إحدى محافظات، قرى او هجر المنطقة

أي من الطرق التالية تساعد بطريقة فعالة في الحد من الإصابة بفايروس كورونا المستجد (كوفيد -١٩)
استخدام غسول الفم

غسل اليد قبل ملامسة الوجه، الأنف أو العينين
تناول مضادات حيوية
الحصول على تطعيمات الأنفلونزا الموسمية
تناول فيتامينات لتقوية المناعة

هل تعتقد ان الفايروس يستطيع البقاء على الأسطح كمقابض الأبواب
نعم
لا

هل تعلم ان هناك طريقة مخصصة يجب مراعاتها أثناء غسل اليدين
نعم
لا

ما مدى تطبيقك للإجراءات الموصى بها من منظمة الصحة العالمية للوقاية من فايروس كورونا مثل غسل اليدين
أطبقها دائما
أطبقها احيانا
لا أطبقها
طبقتها بعد ظهور الحالات في المملكة

عند الخروج من المنزل، هل تلبس القفازات
نعم دائما
لا
نعم احيانا
لم اغادر المنزل

هل تستخدم الكمامات المتاحة عند مدخل السوبرماركت
نعم دائما
نعم احيانا
لا ، لعدم توفرها
لا ، لعدم رغبتي في استخدمها
لم اغادر المنزل

هل تطبق السلام بدون مصافحة " السلام نظر"
نعم اطبقها دائما
نعم اطبقها غالبا
أجد صعوبة ولكني احاول
لا اطبقها لأني أرى فيها تقليل من تقدير الشخص الاخر
لا اطبقها لعدم تقبل المجتمع لهذه الظاهرة
لا اطبقها لاني اعتقد انها غير ضرورية للحد من انتشار العدوى

عند العودة الى المنزل خلال فتره الحجر المنزلي
اغسل يدي بالماء والصابون لمدة ٤٠ ثانية
استخدم المعقم لمدة ٥ ثوان
اغسل يدي بالماء والصابون اقل من ٤٠ ثانية
استخدم المعقم لمدة ٢٠ ثانية
لا اغسل يدي ولا استخدم المعقم
استخدم المعقم احيانا وأحيانا اغسل يدي

منذ بداية انتشار فايروس كورونا المستجد (كوفيد-١٩) تم تغير

طريقة غسل اليدين

زيادة عدد مرات غسيل اليدين

طريقة غسل اليدين مع زيادة عدد مرات الغسل

لم يتغير شي في هذا الجانب
