# Supplementary material for: Knowledge and practice of personal protective measures during the COVID-19 pandemic: A cross-sectional study in Saudi Arabia
Source: PLoS One. 2020 Dec 11;15(12):e0243695. doi: 10.1371/journal.pone.0243695 (PMC7732079; doi:10.1371/journal.pone.0243695)
Supplement: S1 Table — (PDF) [file pone.0243695.s002.pdf]

S2 Table. Mean rank of total scores in regards to knowledge questions

Demographic characteristics		Mean Rank	*p* value
**Gender**	Male	2480.63	<0.001*
	Female	2604.61	
**Age group**	18-27 years #	2525.49	0.009*
	28-37 years #	2542.5	
	38-47 years	2624.71	
	Above 47 years	2530.49	
**Marital status**	Single	2542.15	0.325
	Married	2554.04	
	Divorced	2626.4	
**Educational level**	Secondary and below $	2432.57	<0.001*
	Diploma	2548.13	
	Bachelor	2587.26	
	Master and PhD	2654.37	
**Income**	Less than 3000 SR Ø	2508.89	0.001*
	3000-5999 SR	2523.54	
	6000-10999 SR	2563.15	
	11000-15999 SR	2601.81	
	16000-20000 SR	2621.18	
	Above 20000 SR	2659.43	
**Employment**	Unemployed	2545.37	0.71
	Student	2496.62	
	Employed	2583.88	
	Entrepreneur	2617.14	
	Retired	2541.87	
	Other	2471.28	
**Geographical regions**	West	2559.89	0.056
	Middle	2577.32	
	East	2595.37	

	North	2505.36	
	South	2454.92	
**Do you live in**	City	2556.85	0.175
	Village	2494.97	

* statistical significance; # compared to 38-47, $ compared to diploma, bachelor, master and PhD, Ø compared to above 20000 SR.
